# Preparation of ZnO Nanoparticle/Acrylic Resin Superhydrophobic Coating via Blending Method and Its Wear Resistance and Antibacterial Properties

**DOI:** 10.3390/ma14143775

**Published:** 2021-07-06

**Authors:** Changquan Li, Chen Wang, Ziang Li, Zhenjun Cao, Yu Xie, Mingshan Xue, Jinsheng Zhao

**Affiliations:** 1 School of Materials and Engineering, Jiangsu University of Technology, Changzhou 213001, China; lichangquan@163.com (C.L.); wangchen@163.com (C.W.); liziang@163.com (Z.L.); 2 College of Environment and Chemical Engineering, Nanchang Hangkong University, Nanchang 330063, China; caozhenjun@163.com (Z.C.); xuemingshan@163.com (M.X.); 3 College of Chemistry and Chemical Engineering, Liaocheng University, Liaocheng 252059, China

**Keywords:** ZnO nanoparticle, acrylic resin, superhydrophobic coating, anti-biofouling, aluminum substrate

## Abstract

Herein, a facile method for the preparation of an acrylic resin-based superhydrophobic coating is provided. Firstly, ZnO nanoparticles were modified with silane to obtain hydrophobic ZnO, which was then homogeneously blended with acrylic resin. Subsequently, the mixture was sprayed on an aluminum sheet to form a cured coating. The surface composition and morphology of the coating were characterized using X-ray diffraction (XRD), Fourier transform infrared spectroscopy (FTIR), and scanning electron microscopy (SEM). The hydrophobicity, wear resistance, and antibacterial properties of the prepared samples were tested. The optimized hydrophobicity was achieved with 10 wt% modification agent and resin-to-ZnO mass ratio of 1:4, exhibiting contact and sliding angles of 168.11° and 7.2°, respectively. Wear resistance was insufficient with a low resin content, while it grew with the increase in the resin content. However, when the resin content was excessively high, the hydrophobicity was reduced because the resin could wrap the modified ZnO nanoparticles and decrease the number of hydrophobic groups on the surface. Compared with the pure acrylic resin coating, the ZnO nanoparticle/acrylic resin superhydrophobic coating demonstrated a significant enhancement in the antibacterial properties.

## 1. Introduction

Owing to their unique properties, superhydrophobic coatings can be used in self-cleaning [1,2,3,4,5], anti-corrosion [6,7,8], anti-icing [8,9,10], and oil–water separation [10,11,12], suggesting great potential in a broad range of applications [13,14,15,16,17,18,19,20,21,22]. ZnO nanoparticle (nano-ZnO) has strong anti-bacterial properties because of its low volume and high surface activity [23,24,25]. Hsieh et al. [26] successfully prepared a superhydrophobic coating using a composite of ZnO and fluorinated polymer. Spasova et al. [21] reported the superhydrophobic surfaces composed of ZnO nanoparticles-containing polyvinylidene fluoride (PVDF) and polyvinylidene fluoride-hexafluoro propylene (PVDF-HFP) via the electrostatic spinning method [27]. Chemicals with low surface energies are commonly used in the preparation of hydrophobic materials, including siloxanes, fluorides, and long-chain alkanoic acids, which can form covalent bonds with nano-ZnO by breaking their chemical bonds and chains [28,29,30]. After this modification, the microstructure of nano-ZnO does not change and nano-ZnO becomes superhydrophobic while retaining its original semiconductor (optical, electric, and magnetic) properties. Guo et al. [31] prepared a transparent thin film composed of ZnO-nanorod array using the hydrothermal method. Sakai et al. [32] fabricated a ZnO thin film on the Si substrate via the sol–gel method, and then obtained a superhydrophobic ZnO thin film after drying.

Herein, a facile method for the preparation of an acrylic resin-based superhydrophobic coating is reported. Firstly, nano-ZnO was modified with organosilane to obtain hydrophobic nano-ZnO. The suspension of hydrophobic nano-ZnO was homogeneously blended with acrylic resin. Then, the mixture was sprayed onto an aluminum sheet to form a cured coating. The surface composition and morphology of the coating were characterized using X-ray diffraction (XRD), Fourier transform infrared spectroscopy (FTIR), and scanning electron microscopy (SEM). The effects of modifier content, mass ratio between acrylic resin, and nano-ZnO on the hydrophobicity of the samples were studied, and the wear resistance was measured. The potential applications of the coatings prepared in the present study may include the surface protection for marine vessels and offshore pipelines, and bottom protection for various marine facilities.

## 2. Experimental

### 2.1. Preparation of Superhydrophobic Coatings

**Figure 1:**
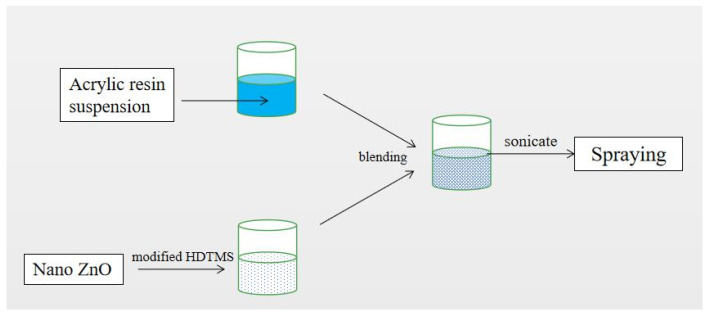
Flow chart of preparation of superhydrophobic coating of acrylic resin via the blending method.

The aluminum sheet (50 × 50 × 50 mm^3^) was sanded and polished with 600-mesh sandpapers, rubbed five times from eight different directions every minute for 10 min, followed by rinsing in de-ionized water for 1 min. The acrylic acid primer and corresponding diluent (Changshu Fangta Paint Co. Ltd., Changshu, China) were mixed to form the suspension, as per the instructions. The as-polished aluminum sheet was placed in a fume hood for the spraying process. The primer mixture was loaded in the sprayer and the distance between the sprayer nozzle and aluminum sheet was maintained at 10 cm [33]. The duration for spraying was 1 min and the sprayed sheet was dried at room temperature for over 6 h. The primer can enhance the binding strength, fullness, alkali resistance, and anti-corrosion, and ensure the homogeneous formation of an acrylic acid coating. The nano-ZnO (99.9%, Beijing Huanqiujinding Tech Co. Ltd., Beijing, China) with sizes of 30 nm and 90 nm were mixed in the ratio of 1:1 and added into the solution of acetone (analytical reagent, Xilong Chemistry Co. Ltd., Shantou, China) and water (3:1 *v*/*v*), reaching a weight percentage of 3%. Hexadecyltrimethoxysilane (HDTMS, chemical reagent, Shanghai Dibo Biology Tech Co. Ltd., Shanghai, China) of different weight % (2, 4, 6, 8, and 10%) was added as surfactant to nano-ZnO nanoparticles. The mixture was stirred using a magnetic stirrer and maintained at 60 °C for 3 h to obtain a ZnO suspension. A suspension of acrylic topcoat (Changshu Fangta Coating Chemistry Co. Ltd., Changshu, China) and ZnO was prepared in the weight ratio of 0.5:2, 1:2, 1.5:2, 2:2, 2.5:2, and 3:2, as per instructions. After curing at room temperature for 30 min, the nano-ZnO suspension was added into the topcoat suspension, and the mixture was stirred and ultrasonicated for 10 min before loading into the sprayer. After spraying on the primer layer for 1 min, the coating was cured at room temperature for 6 h to obtain superhydrophobic nano-ZnO/acrylic resin composite coating. A schematic of the preparation process is presented in Figure 1.

**Figure 2:**
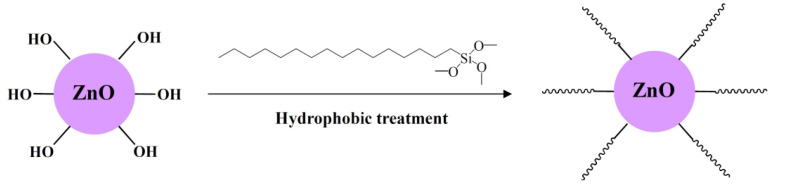
Surface modification mechanism of ZnO.

The surface modification mechanism of ZnO is demonstrated in Figure 2. After hydrolysis, the methoxyls of HDTMS were replaced by the hydroxyls. As the surface of ZnO particle has several hydroxyls, it can form hydrogen bonds with hydrolyzed HDTMS. By changing the reaction conditions, the hydrolyzed HDTMS can react with hydroxyls on the ZnO surface and dehydrate, forming a network structure. Meanwhile, -CH_3_ and -CH_2_- hydrophobic groups were grafted onto the ZnO surface. These hydrophobic groups can largely enhance the stability and surface roughness, as well as the hydrophobicity.

### 2.2. Structure and Morphology Testing and Characterization

XRD was performed using an X-ray diffractomer (Bruker D8 ADVANCE, Bruker, Karlsruhe, Germany) with a Cu-Kα source at the scan rate of 4° min^−1^ in the range of 20–80°. An environmental SEM (FEI Quanta 200, Thermo Fisher, Waltham, MA, USA) and field-emission SEM (FEI Nova Nano FE-SEM, Thermo Fisher, Waltham, MA, USA) were used in this study. The acceleration voltage was 15 kV and the chamber was vacuumed during imaging. The molecular structure and chemical composition were characterized using a FTIR spectrometer (Bruker Vector 22, Bruker, Karlsruhe, Germany).

**Figure 3:**
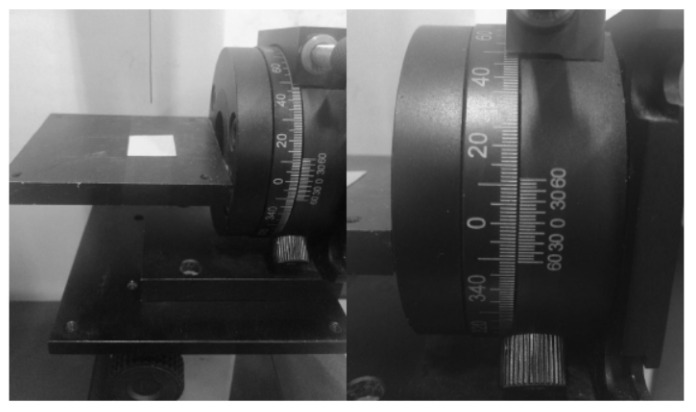
Test rotary table for rolling angle.

The hydrophilicity was characterized by measuring the contact and rolling angles using a contact angle meter (Krűss DSA20, Krűss Company, Hamburg, Germany). During the measurements, a small amount of water was dropped on the sample surface and the contact angle was measured with the meter. By flipping the sample stage, when the droplet rolled off, the rolling angle could be measured and recorded with the vernier angle caliper, as shown in Figure 3. 9 μL of water was used for measurements and the average contact and rolling angles for each sample were averaged after five measurements.

**Figure 4:**
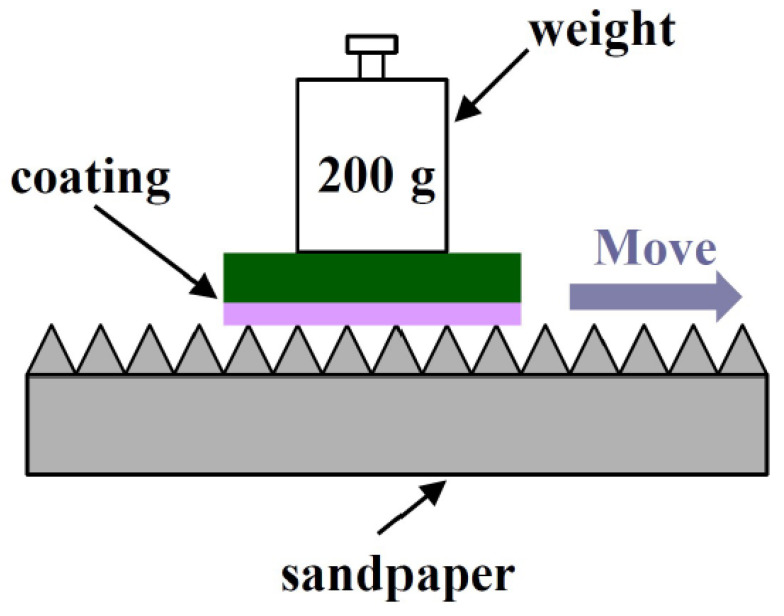
Schematic diagram of the anti-wear experiment.

The measurement for wear resistance of the sample was performed by sanding the sample with a 220-mesh sandpaper, as shown in Figure 4. The relative speed was 5 cm s^−1^, while the pressure was 1000 Pa. The contact and rolling angles were recorded after sanding every five times.

### 2.3. Antibacterial Testing

#### 2.3.1. Specimen Preparation and Pretreatment

The superhydrophobic samples and comparison samples (coated with pure acrylic resin) were cut into five identical 20 × 20 mm^2^ specimens. The test results of five specimens were averaged. The surface was rinsed with 70 wt% ethyl alcohol, rinsed with de-ionized water for 5 min, and then dried naturally.

#### 2.3.2. Bacteria Inoculation

The superhydrophobic samples and comparison samples were used for the test. The gram-positive bacteria *S. aureus* was used as the testing bacteria. The agar medium for the test consisted of peptone (5 g), beef extract (3 g), and sodium chloride (5 g) in 1000 mL distilled water with a pH value of 7. Finally, agar (15 g) was added to the solution. The medium was sterilized in a autoclave for 30 min under a pressure of 7.0 kg. The standard conditions of 106 CFU/mL (colony forming unit/mL) was utilized for antibacterial assay. This medium was transferred into sterilized Petri dishes. The bacteria species (50 µL) cultures were spread on the solid surface of the media after solidification of the media. Over this inoculated petri dish, small pieces of the samples and untreated samples were placed and incubated for two days at 37 °C in an incubation chamber to observe the inhibition zone. All the experiments were performed in triplicate to confirm their reproducibility.

#### 2.3.3. Colony Count

The inoculated samples were eluted after culturing and the elution was transferred in an Erlenmeyer beaker. After stirring and diluting, the elution was transferred to Petri dishes to inoculate bacteria. The Petri dishes were placed in an incubator for 24 h before counting colonies.

#### 2.3.4. Antibacterial Rate Calculation

The antibacterial rate was calculated from Equation (1):

(1) $$R=\frac{A-B}{A}\times 100\%$$

where *R* is the antibacterial rate, *A* is the colony number for the comparison sample after a 24 h of incubation time, and *B* is the colony number for the superhydrophobic sample after a 24 h of incubation time.

## 3. Results and Discussion

### 3.1. FTIR Analysis

**Figure 5:**
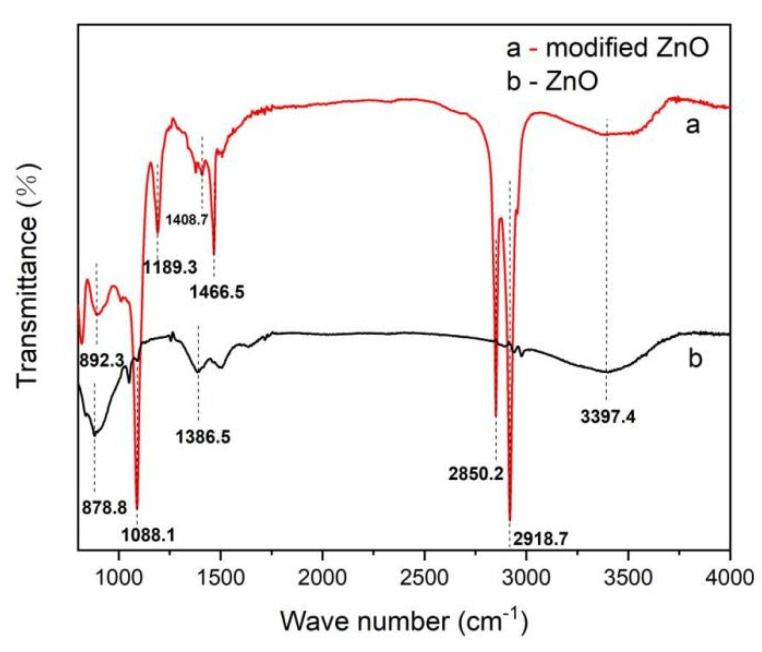
Infrared spectra of nano-ZnO before and after modification.

The FTIR spectra of nano-ZnO and modified nano-ZnO are shown in Figure 5, respectively. Absorption peaks in the range of 1088.1 cm^−1^ can be observed in both the spectra, which is caused by the stretching and bending vibrations of hydroxyls or bridged hydroxyls on the surface of nano-ZnO. In the ambient environment, water molecules can be adsorbed by the surface of nano-ZnO, which can then decompose to form these adsorbed hydroxyl groups. The absorption peaks at 878.8 and 1386.5 cm^−1^ are the characteristic and vibration peaks of Zn–O bonds, respectively.

Compared with the FTIR spectrum of nano-ZnO, several significant changes were observed in the spectrum of modified nano-ZnO. The peak at 1466.5 cm^−1^ resulted from the antisymmetric bending vibration of methyls and methylenes [34]. The new peak at 1189.3 cm^−1^ can be attributed to the vibration of the ester groups of the cross-linking agent. The peak at 2918.7 cm^−1^ was due to the stretching vibration of C-H bond (anti-symmetric vibration) in the CH_3_ unit, and the peak at 2850.2 cm^−1^ was due to the stretching vibration of C-H bond (symmetric vibration) in the CH_3_ unit. The peak at 1408.7 cm^−1^ was due to the in-plane bending vibration of C-H bond. The peak at 892.3 cm^−1^ was due to the stretching vibration of Zn–O–Si bonds[z]. This is due to the hydrolysis of HDTMS, which converts Si–O–CH_3_ to Si–OH, and subsequently reacts with –OH on the surface of nano-ZnO. The reaction equations are as follows:

ZnO + H_2_O = ZnO-OH + H (2)

CH_3_(CH_2_)_15_Si(OCH_3_)_3_ + 3H_2_O = CH_3_(CH_2_)_15_Si(OH)_3_ + 3CH_3_OH (3)

3ZnO-OH + CH_3_(CH_2_)_15_Si(OH)_3_ = CH_3_(CH_2_)_15_Si(OZn)_3_ + 3H_2_O (4)

### 3.2. XRD Analysis

**Figure 6:**
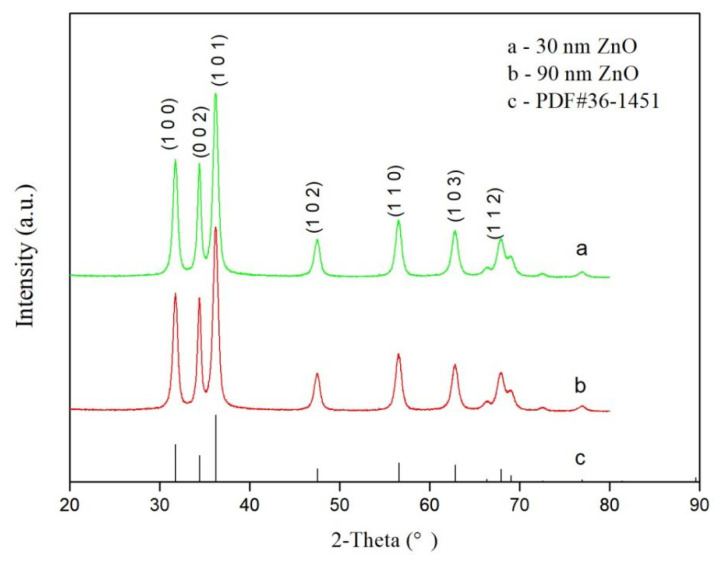
X-ray diffraction (XRD) patterns of nano-ZnO of two sizes.

The XRD patterns of nano-ZnO with particle sizes of 30 nm and 90 nm are shown in Figure 6. Both patterns are consistent with the PDF standard files (JCPDS 36-1451), suggesting that nano-ZnO is a hexagonal crystalline. Moreover, no impurity peak was found in the patterns besides the characteristic peaks of ZnO; therefore, the purchased ZnO had a high purity without the addition of impurity particles. The diffraction peaks were relatively high and narrow, especially for those of 90 nm nano-ZnO, indicating that both nano-ZnO particles had good crystallinity. The calculation based on the full width at half height indicates that the particle size of 90 nm nano-ZnO was larger than the one with 30 nm.

### 3.3. Morphology and EDS Analysis

**Figure 7:**
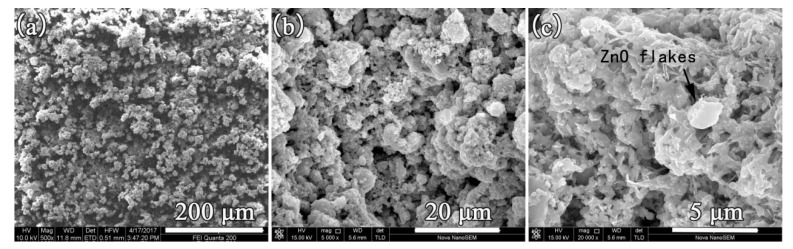
FE-scanning electron microscopy (SEM) image of a sample at different magnifications. (**a**): 500×; (**b**):5000× (**c**): 20,000×.

**Figure 8:**
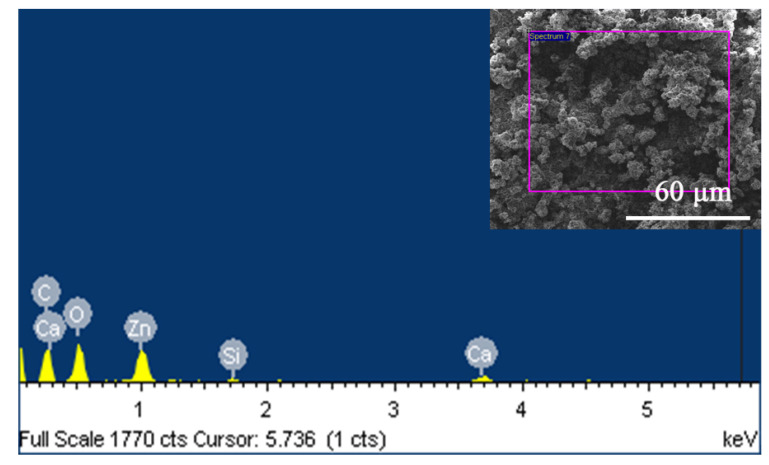
EDS diagram of superhydrophobic samples.

Figure 7 presents the FE-SEM images of the as-prepared superhydrophobic samples at various magnifications. The image at low magnification (Figure 7a) shows multiple small, coral-like lumps uniformly distributed on the surface of the superhydrophobic sample, suggesting that the spraying process was homogeneous across the substrate surface. At a high magnification (Figure 7c), the shape of ZnO particles was flake-like, which can be found on the surface of acrylic resin, embedded into the resin, or wrapped by the resin. The abundant fine pores on the surface with a good roughness are favorable for hydrophobicity. Figure 8. shows the EDS result of the as-prepared superhydrophobic sample. The elements found on the surface were C, O, Zn, Ca, and a small amount of Si. The origin of Ca in the EDS diagram is the presence of the CaCO_3_, as the filler in acrylic resin. The result agrees with the content of HDTMS used for the modification of ZnO, further indicating the successful grafting of HDTMS on the surface of ZnO.

### 3.4. Cross-Section Morphology

**Figure 9:**
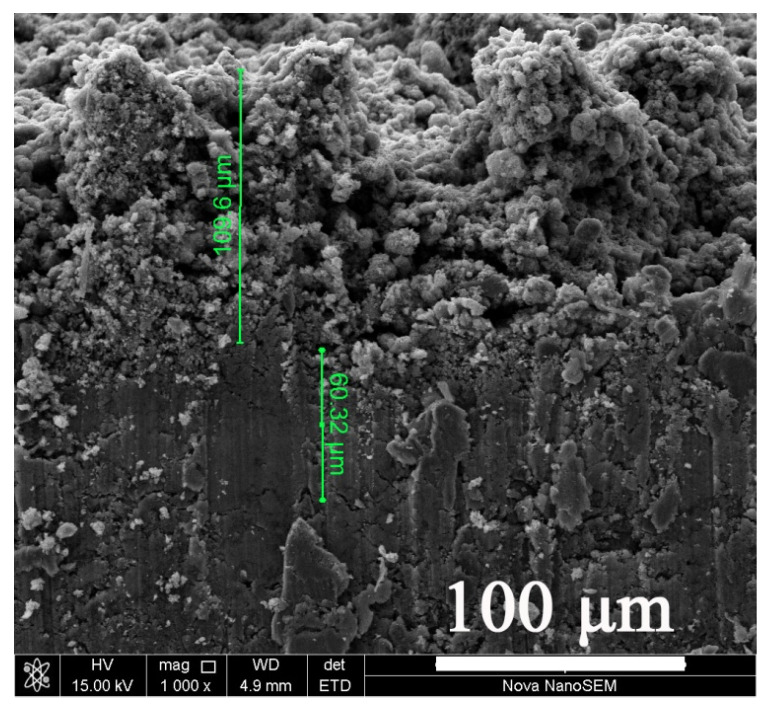
SEM cross-sectional image of a superhydrophobic sample.

The cross-sectional SEM image of the as-prepared superhydrophobic sample is shown in Figure 9. A clear three-layered structure was found on the cross-section. The upper layer with the thickness of 106 μm was a mixture of nano-ZnO and acrylic resin decorated with several fine particles, signifying a relatively high roughness. The medium layer with the thickness of 60.32 μm was the primer and significantly smoother than the upper layer. The lower layer was aluminum sheet and all three layers displayed good binding with each other.

### 3.5. Effect of Surfactant on Hydrophobicity

**Figure 10:**
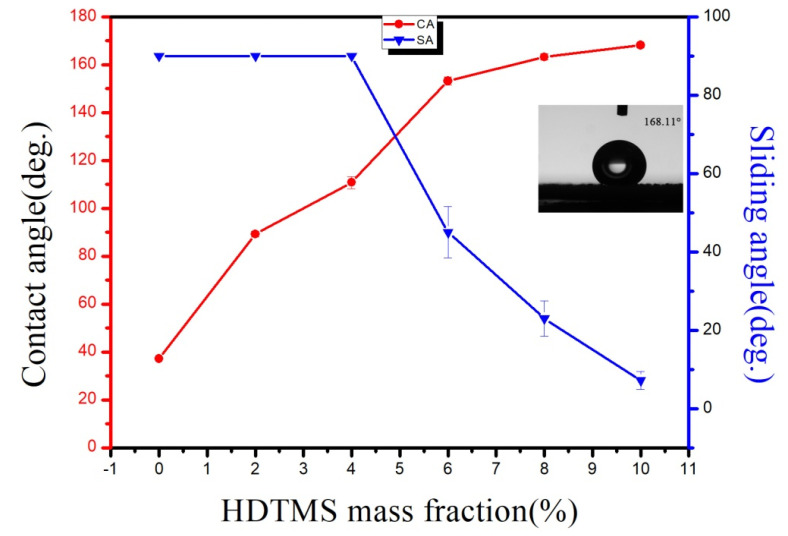
Wettability of samples modified with different mass fractions of HDTMS.

The change in surface wettability of the samples modified with various mass fractions of HDTMS is presented in Figure 10. The mass ratio of acrylic resin to nano-ZnO was maintained at 1:4. With the increase in HDTMS content, the contact angle of the sample increased while the sliding angle decreased. Without the addition of a surfactant, the contact angle was merely 37.21°. When the HDTMS content increased to 10%, the improvement reached the highest point with a contact angle of 168.11° and a sliding angle of 7.2°. However, the sliding angle was still relatively high as a high surface roughness leads to a lag in the variation of sliding angles.

### 3.6. Effect of Weight Ratio of Acrylic Resin to ZnO on Hydrophobicity

**Figure 11:**
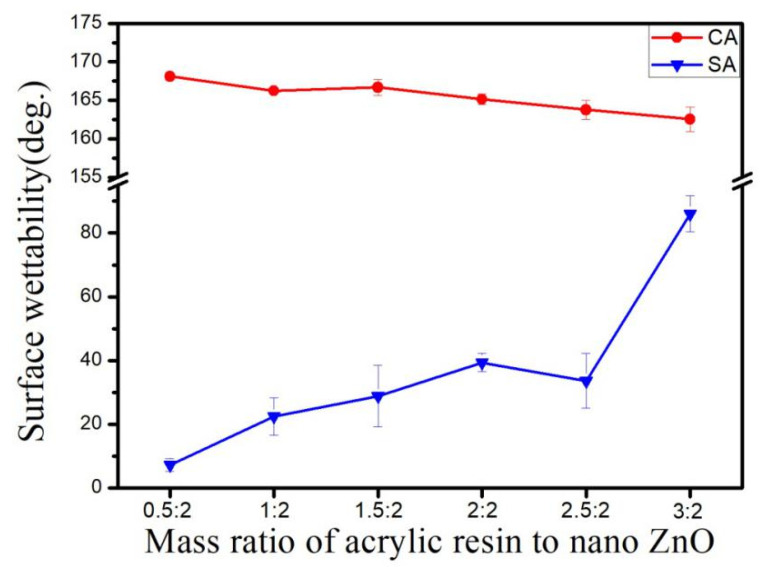
Sample wettability of different acrylic resin-to-ZnO mass ratio.

The effect of the weight ratio of acrylic resin to ZnO on hydrophobicity is shown in Figure 11. At the ratio of 0.5:2, the contact and sliding angles of the sample were 168.11° and 7.2°, respectively. When the ratio increased to 3:2, the contact angle decreased to 162.54° while the sliding angle increased to 86°. The angle variations indicate that the hydrophobicity of the sample was reduced with the increase in acrylic resin because the modified nano-ZnO was more wrapped with resin. The exposed surface of nano-ZnO was reduced; hence, the hydrophobicity decreased [35]. When the ratio of acrylic resin to ZnO is 3:2, there is a sharp increase in the sliding angle, the main reason being that, with the increase of resin content, more nano ZnO particles are wrapped by resin, resulting in the decrease of exposed nano ZnO particles, and then leading to the decrease of hydrophobicity and the increase of sliding angle.

### 3.7. Wear Resistance

**Figure 12:**
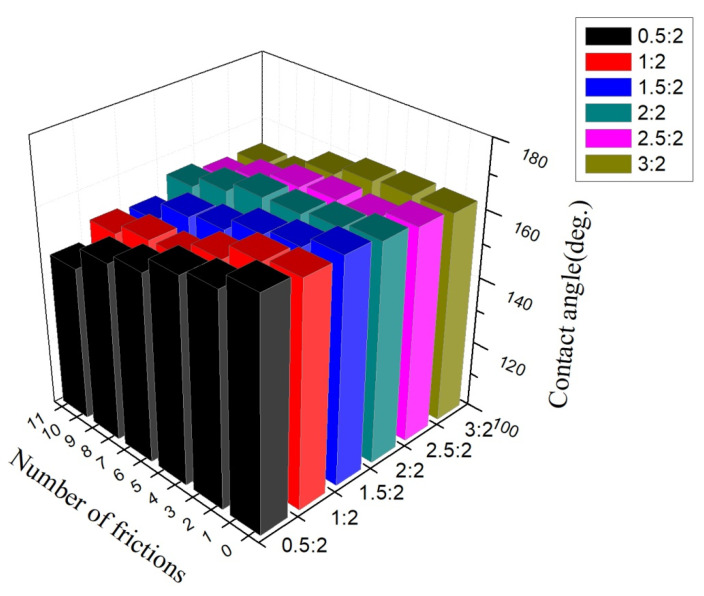
Relationship between the number of frictions and wettability of samples prepared with various mass ratios of resin to ZnO.

**Figure 13:**
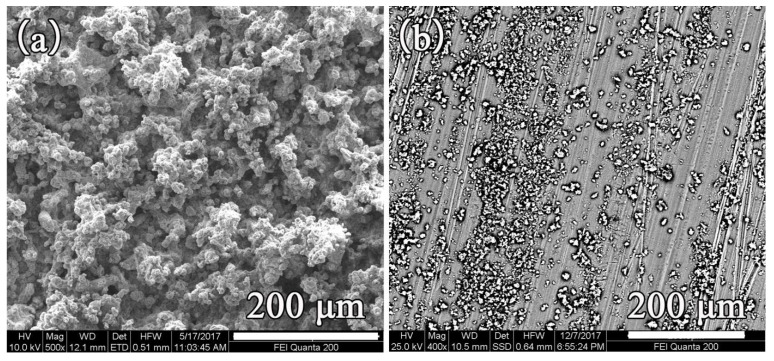
SEM image of sample prepared with resin-to-ZnO ratio of 1:4 (**a**) before and (**b**) after sanding 10 times.

Figure 12 presents the relationship between the times of sanding and wettability of the samples prepared by various mass ratios of resin to ZnO. As shown in Figure 12, with the increase of the friction times, the hydrophobicity of all the six samples decreased, but with different downward trends. A greater decrease was found in the sample with a lower ratio of acrylic resin. With higher content of acrylic resin, there was only a slight downward trend in hydrophobicity. This is because, with larger ratios of resin, the binding force within resin was stronger, which can lead to formation of a network structure, enhancing wear resistance in the process. All the samples shown in Figure 12 were only sanded 10 times and the contact angle was already decreased to 146.06°, which cannot meet the requirement of superhydrophobicity. Figure 13 presents the SEM image of the sample prepared with a resin-to-ZnO ratio of 1:4 after sanding 10 times. The particles on the surface were mostly exfoliated and only a small number of ZnO particles were found on the surface. Nano-ZnO inhibits resin forming films, which increases the interstices between molecules, causing the binding strength of resin to decrease. Hence, the surface shows poorer wear resistance, leading to a reduction in roughness and hydrophobicity [35].

### 3.8. Antibacterial Properties

The antibacterial properties of the superhydrophobic sample and comparison samples were analyzed based on the standard method for inorganic nanomaterials [36]. The comparison sample was the pure acrylic resin coating. The colony counts after the bacterial inoculation on the sample surface are listed in Table 1. The results indicate that the antibacterial rate of the superhydrophobic sample was 100%, while that of the comparison sample was 0. The antibacterial data suggest that the superhydrophobic nano-ZnO/acrylic resin coating possesses excellent antibacterial properties, while the pure acrylic resin coating hardly had this property. This is owing to the dissolving Zn^2+^ ions on the surface of the superhydrophobic nano-ZnO/acrylic resin coating, which can damage the bacterial structure. Meanwhile, the superhydrophobic coating can shield water because of the air-containing layer, reducing the adhesion of bacterial proteins on the surface and partly contributing to the antibacterial properties of superhydrophobic coating. Therefore, the antibacterial behavior of the nano-ZnO/acrylic resin superhydrophobic coating can be accredited to the coupling effect of the dissolved Zn^2+^ ions and the superhydrophobic insulation [36,37]. The superhydrophobicity of the surface is usually covered with organic film, which will greatly improve the hydrophobicity of the surface of the material. However, the film formed by organic matter is often of poor mechanical strength, and high temperature or external force wear will lead to the damage of organic film. Thus, surface superhydrophobicity and mechanical stability are mutually exclusive properties for the organic/inorganic hybrid. The development or adoption of new organic resin materials may be one of the trends in the future.

## 4. Conclusions

The as-prepared nano-ZnO/acrylic resin superhydrophobic coating possessed a good hydrophobicity. The morphological characterization shows that the micro/nano structure of the composite was uniformly distributed on the sample surface, suggesting that the spraying process was homogeneous. The preparation method is simple and can be suitable for large-scale manufacturing. The effect of the surfactant content and the mass ratio of acrylic resin to nano-ZnO on the hydrophobicity of the coating was studied. When the surfactant content was 10 wt% and the ratio of acrylic resin to nano-ZnO was 1:4, and the hydrophobicity was the best with contact and rolling angles of 168.11° and 7.2, respectively. The wear resistance test of the coatings with various acrylic resin ratios showed that the wear resistance was insufficient with a low resin content and increased with the resin content. However, the hydrophobicity of the coating decreased when the resin content was excessive owing to the wrapping of nano-ZnO by the resin, which reduced the number of hydrophobic groups on the surface and worsened the hydrophobicity of the coating. The antibacterial tests showed that, compared with the pure acrylic resin coating, the superhydrophobic nano-ZnO/acrylic resin coating possessed superb antibacterial properties.

## Figures and Tables

**Table 1:** Antibacterial properties of nano-ZnO/acrylic resin and pure acrylic resin.

Sample	Colony Counts	Antibacterial Rate
nano-ZnO/acrylic resin superhydrophobic coating	0	100%
Comparison sample (pure acrylic resin coating)	57	0

## Data Availability

All the data is available within the manuscript.
